# Application of Polymer-Embedded Tetrabutylammonium Bromide (TBAB) Membranes for the Selective Extraction of Metal Ions from Aqueous Solutions

**DOI:** 10.3390/ma16165721

**Published:** 2023-08-21

**Authors:** Beata Pospiech

**Affiliations:** Faculty of Production Engineering and Materials Technology, Czestochowa University of Technology, Armii Krajowej 19, 42-200 Czestochowa, Poland; beata.pospiech@pcz.pl

**Keywords:** polymer inclusion membranes, iron(III), nickel(II), cobalt(II), lithium(I), ionic liquids, tetrabutylammonium bromide (TBAB), separation processes

## Abstract

The selective extraction of metals from aqueous solutions is a very important stage in the hydrometallurgical processing of metallic waste. Leach solutions are usually a multicomponent mixture. The main impurity of aqueous solutions obtained after leaching using inorganic acids is iron. In this work, the membrane separation of iron(III) from nickel(II), cobalt(II), and lithium(I) was studied. The facilitated transport of metal ions using polymer inclusion membranes (PIMs) with tetrabutylammonium bromide (TBAB) as an ion carrier under various conditions was analyzed in detail. Several factors, such as the ion carrier concentration in the membrane as well as the effect of the inorganic acid concentration in the source/receiving phases on the kinetic parameters, were investigated. The results show that ionic liquid TBAB is a very selective ion carrier of Fe(III) towards Ni(II), Co(II), and Li(I).

## 1. Introduction

The selective removal of metal ions from aqueous solutions using polymer inclusion membranes (PIMs) can be an effective method of separation.

Membrane techniques, including PIMs, are studied for various applications, e.g., the hydrometallurgical recovery of metals from electronic waste. The synthesis of PIMs is very easy and requires small amounts of reagents. A suitable carrier guarantees the high selectivity of such a membrane. Moreover, PIMs are more stable than supported liquid membranes (SLMs). Therefore, polymer membranes have attracted a great deal of attention recently.

The separation of metal ions from aqueous solutions is one of the most important stages in the hydrometallurgical process of metal recovery. The proposal to use polymer membranes for the separation of metal ions is noteworthy due to the number of advantages of this method, such as the low cost of producing the membranes and the simple design of the system consisting of a source phase/a membrane/and a receiving phase.

PIMs consist of an ion carrier/extractant immobilized within a polymer matrix (i.e., cellulose triacetate CTA, poly(vinyl chloride) PVC, poly(vinylidene fluoride-*co*-hexafluoropropylene) PVDF-HFP) [1,2]. The choice of ion carrier depends on the metal species present in the aqueous solutions [3]. An additional substance applied for PIM synthesis is a plasticizer, which gives the membrane softness and flexibility (i.e., 2-nitrophenyl octyl ether (NPOE), 2-nitrophenyl pentyl ether (NPPE), etc.) [3,4]. Various types of extractants/ion carriers are used for membrane synthesis. It depends on the form of the metal species present in the aqueous solution [1]. The transport of metal ions is related to the chemical composition of the polymer membrane as well as the composition of the aqueous solutions. Further improvement in PIM technology is necessary as it may significantly contribute to the commercialization of this type of membranes. Modifications in the composition of membranes by means of additional new components may have a positive effect on metal ion migration. Currently, we can use many research techniques to characterize the surface of membranes and study their structure, e.g., atomic force microscopy (AFM), scanning electron microscopy (SEM), etc. The aspect of the membrane structure is very important because it allows many phenomena related to membrane transport to be explained [1,2,3,4].

Many authors have proposed various mechanisms of the facilitated transport of metal ions, such as (a) carrier-diffusion, (b) fixed-site jumping and mobile-site jumping mechanisms, wherein the extracted species jumps between fixed carrier sites [2,5,6,7,8]. Owing to the very different proposals, Hu et al. [5] reported that the mechanism for the transport of metal ions through PIMs is still open to be verified, especially for membranes containing new ion carriers, such as ionic liquids (ILs) called “green solvents” [9]. They have many advantages such as nonvolatility, high thermal stability, and extracting properties [10]. ILs are used both as extractants and ion carriers of metal ions [9,10,11,12,13,14,15,16].

**Figure 1:**
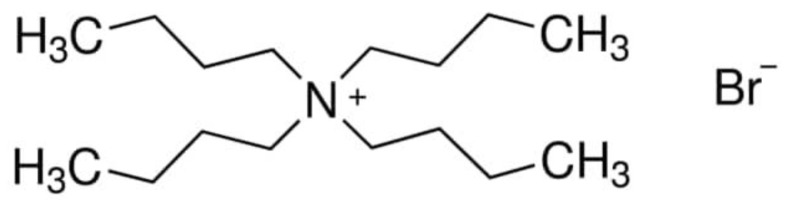
Structure of tetrabutylammonium bromide (TBAB) [18].

Tetrabutylammonium bromide (TBAB) is a quaternary ammonium salt that can dissolve in both aqueous as well as in organic solvents [17]. This reagent is inexpensive, nontoxic, noncorrosive, and environmentally friendly. Its melting point is over 100 °C and this compound is classified as an ionic liquid. Figure 1 presents the structure of TBAB [18]. As can be seen, this salt contains organic cations and inorganic anions. This compound was used as an extractant of Fe(III) [19], Cr(VI) [20,21], and anionic dyes (e.g., golden yellow), etc. [22].

Taking into account the extracting properties of TBAB and the existing need for the selective separation of metal ions from various leach liquors (e.g., the leach liquor of spent lithium-ion batteries (LIBs)), the main aim of this work is to develop an alternative separation technique of Fe(III) from Ni(II), Co(II), and Li(I) from aqueous solutions using PIMs with TBAB as the ion carrier.

## 2. Materials and Methods

### 2.1. Inorganic Chemicals

The inorganic chemicals, i.e., iron(III) chloride (FeCl_3_), cobalt(II) chloride (CoCl_2_·6H_2_O), nickel(II) chloride (NiCl_2_·6H_2_O), lithium(I) chloride (LiCl), hydrochloric acid (HCl), and sulfuric acid (H_2_SO_4_) were of analytical grade and were purchased from POCh (Gliwice, Poland).

### 2.2. Organic Chemicals

The organic chemicals, i.e., tetrabutylammonium bromide (TBAB) (Acros Organics, Antwerp, Belgium, purity ≥ 98.0%), cellulose triacetate (CTA), o-nitrophenyl octyl ether (NPOE), and dichloromethane were of analytical grade (Sigma-Aldrich, Poznan, Poland) and used without further purification.

### 2.3. Synthesis of Polymer Inclusion Membranes (PIMs)

PIMs were prepared similar to reported in earlier papers [11,12,23]. A mixture containing appropriate amounts of CTA, TBAB, and NPOE in dichloromethane (POCh, Gliwice, Poland) was prepared. This solution was poured into a Petri dish. The dichloromethane was evaporated and the resulting membrane was separated by immersion in distilled water.

### 2.4. Transport of Metal Ions Experiments

The transport experiments were conducted in a similar manner as reported in earlier papers [11,12,23]. The membrane module was used for the transport of Fe(III), Ni(II), Co(II), and Li(I) from the source phase across PIM into the receiving phase. The membrane separated the two phases. Both the aqueous phases were pumped with a peristaltic pump (PP1B-05A, Zalimp, Poland). The volumes of the source and receiving phases were 100 cm^3^, respectively. The source phase contained 0.01 M Fe(III), 0.01 M Co(II), 0.01 M Ni(II), and 0.01 M Li(I) in a hydrochloric acid solution. Sulfuric acid was used as the receiving phase. The effective membrane area was 12.56 cm^2^. The aqueous phases were stirred by magnetic stirrers. The concentration of metal ions was monitored by sampling of the source phase at regular intervals. The concentration of metal ions in the aqueous phases was analyzed by means of a plasma emission spectrometer MP-AES 4200 (Aglilent, Warszawa, Poland). The PIM transport experiments were conducted at room temperature (22 °C). In order to calculate rate constant (*k*), a diagram of the dependence of ln(c/c_i_) vs. time was prepared for each transport of metal ions [23]. When the relationship is linear (which is confirmed by high values of the correlation coefficient (r^2^)), then the kinetics of the PIM transport process are described by a first-order reaction according to the following equation [24]:

(1) $$\ln\left(\frac{c}{c_{i}}\right)=-kt$$

where

*c*: metal ion concentration (mol/dm^3^) in the source phase at some given time;

*c_i_:* initial metal ion concentration in the source phase (M, mol·dm^−3^);

*t*: time (h);

*k*: rate constant (h^−1^).

Permeability coefficient (*P*), initial flux (*J_i_*), and recovery factor (*RF*) of the metal were calculated according to the equations presented in previous works [11,12,23].

(2) $$P=\frac{V}{A}k,$$

where

*V:* volume of the aqueous source phase;

*A*: area of the effective membrane.

(3) $$J_{i}=P\cdot c_{i}$$

(4) $$RF=\frac{c_{i}-c}{c_{i}}\cdot 100\%$$

## 3. Results

### 3.1. Effect of Hydrochloric Acid Concentration on Removal of Fe(III) across PIM from Aqueous Solution

**Figure 2:**
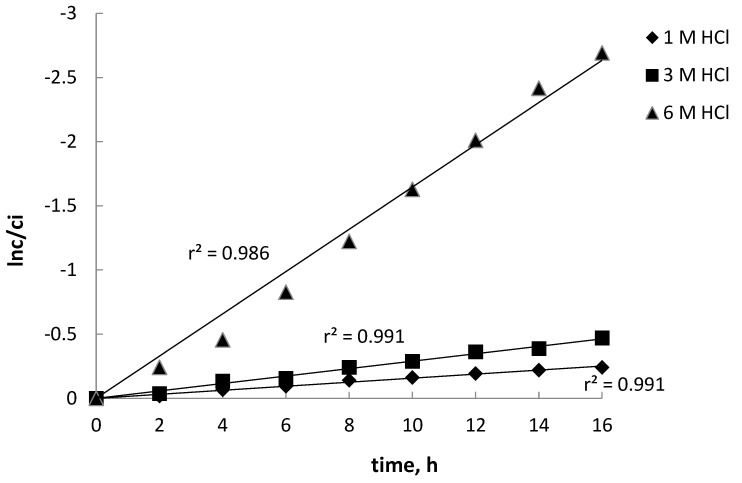
Dependence of ln(c/c_i_) on transport time depending on HCl concentration. Source phase: 0.01 M Fe(III), 0.01 M Ni(II), 0.01 M Co(II), 0.01 M Li(I) in HCl solution. Receiving phase: 1 M H_2_SO_4_. PIM: 35.5% w/w CTA, 49.2% w/w NPOE, and 15.3% w/w TBAB.

One of many parameters affecting the transport of metal ion across a PIM is the concentration of inorganic acid in the source phase containing various metals such as Fe(III), Ni(II), Co(II), and Li(I). In hydrochloric acid solutions, metal can exist as cationic or anionic chlorocomplexes, depending on the chloride ion concentration. For this reason, the effect of the HCl concentration in this phase was studied. This parameter can significantly affect the efficiency and selectivity of the transport of metal ions. The concentration of HCl in the source phase varied between 1.0 mol·dm^−3^ to 6 mol·dm^−3^. Figure 2 shows the plot of ln(c/c_i_) versus the HCl concentration for Fe(III). Other metals (i.e., Ni(II), Co(II), and Li(I)) were not transferred to the 1 M H_2_SO_4_ used as the receiving phase.

**Figure 3:**
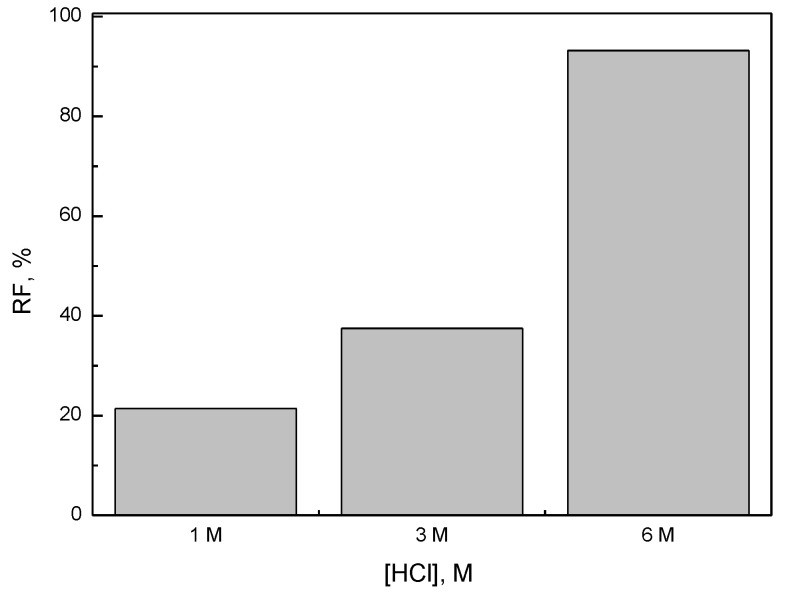
Effect of HCl concentration on recovery factor of Fe(III) (RF, %). Source phase: 0.01 M Fe(III), 0.01 M Ni(II), 0.01 M Co(II), 0.01 M Li(I) in HCl solution. Receiving phase: 1 M H_2_SO_4_, PIM: 35.5% w/w CTA, 49.2% w/w NPOE, and 15.3% w/w TBAB.

Figure 3 presents the dependence of the of the iron(III) recovery (*RF*) versus HCl concentration. Ni(II), Co(II), and Li(I) were not transported to the receiving phase. Thus, it can be concluded that this process is very selective and allows the removal of impurities in the form of iron(III) from aqueous solutions.

Table 1 displays the kinetic parameters of the transport of Fe(III) across PIM depending on the HCl concentration in the source phase. The parameters were the following: rate constant (*k*) and permeability coefficient (*P*) for different HCl concentrations in the receiving phase. Based on the presented data, it can be observed that an increase in acid concentration has a significant effect on rate constant (*k*). Its increase was observed from the value of 0.0157 h^−1^ at a 1 M HCl concentration to the value of 0.1650 h^−1^ at 6 M hydrochloric acid.

**Figure 4:**
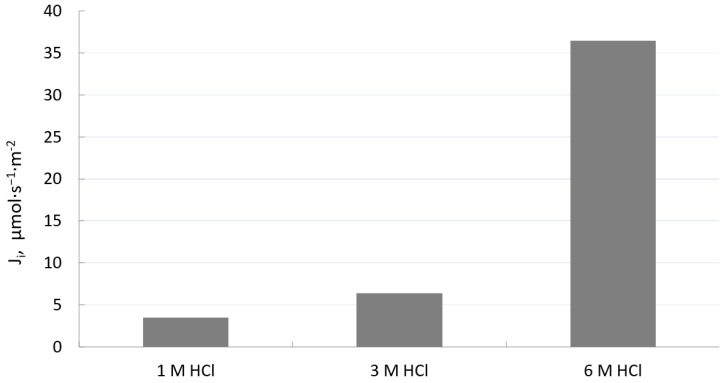
Effect of HCl concentration on initial flux of Fe(III). Conditions are as in Figure 3.

The initial flux is an important parameter characterizing the transport of metal ions from aqueous solutions using PIM. Figure 4 shows the effect of the HCl concentration in the source phase on the initial flux of Fe(III). As can be seen from this figure, initial flux (*J_i_*) for the transport of Fe(III) across PIM with TBAB rises with an increasing hydrochloric acid concentration up to 6 mol·dm^−3^. It can be concluded that a high concentration of acid has a positive effect on the transport efficiency of Fe(III) ions and enables their selective removal.

### 3.2. Influence of PIM Composition on Kinetic Parameter of Transport of Fe(III)

In order to ensure good properties of the membrane, it is first necessary to determine its appropriate chemical composition [25]. This is a key issue. The studied PIM contains CTA as the polymer support, TBAB as the ion carrier, and NPOE is used as the plasticizer. The content of these substances significantly affects the transport and separation properties of PIMs. The content and type of ion carrier and plasticizer are especially prominent. A number of studies have confirmed that one of the best plasticizers is NPOE because polymer membranes containing this plasticizer exhibit much better permeability and are flexible and soft [26]. There are no reports in the literature on the effect of the TBAB concentration on the transport and separation properties of the membrane. Hence, there is a need to determine the optimal concentration of this carrier in PIM. Moreover, it is worth noting that there are no reports in the literature on the application of TBAB as the ion carrier of Fe(III) for the production of PIM. We do not have knowledge about the possibility of using this compound for the synthesis of membranes. Hence, there is a need to determine the optimal concentration of this carrier in the PIM.

The aim of the next series of studies will be the characteristics of the influence of the TBAB concentration on the transport and separation of Fe(III) from 6 M HCl. The concentration of TBAB was varied from 1 to 2 mol·dm^−3^ (based on the volume of plasticizer). Table 2 shows the composition of PIMs, and 1 M sulfuric acid was used as the receiving phase. The source phase contained 0.01 M Fe(III), 0.01 M Ni(II), 0.01 M Co(II), and 0.01 M Li(I) in 6 M HCl.

**Figure 5:**
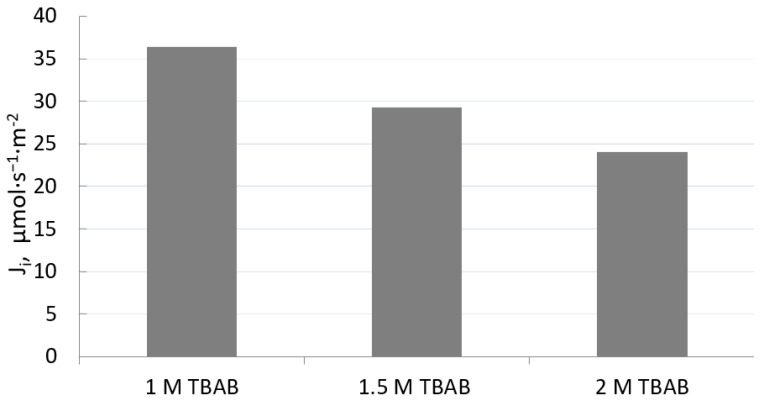
Effect of TBAB concentration in PIMs (in PIM1, PIM2, PIM3)on initial flux (*J_i_*) of Fe(III). Source phase: 0.01 M Fe(III), 0.01 M Ni(II), 0.01 M Co(II), 0.01 M Li(I) in 6 M HCl solution. Receiving phase: 1 M H_2_SO_4_.

The results indicate that only Fe(III) was transported from the source phase, while the Co(II), Ni(II), and Li(I) ions remained in the feed solution. As can be observed from Figure 5, initial flux (*J_i_*) of Fe(III) decreased with an increasing TBAB concentration (based on the volume of plasticizer). It can be concluded that too high a concentration of this carrier is unfavorable for the transport of iron(III) ions. The optimal TBAB concentration in PIM was determined as 1.0 mol/dm^3^. Based on the obtained results, it was found that the optimal composition of the membrane is as follows: 35.5% w/w CTA, 49.2% w/w NPOE, and 15.3% w/w TBAB.

### 3.3. Influence of H_2_SO_4_ Concentration in Receiving Phase on Transport of Fe(III)

Determining the optimal concentration of sulfuric acid in the receiving phase was the next stage of this investigation. H_2_SO_4_ is often used to strip (re-extraction) various metals from the organic phase/membrane phase with good results. Nevertheless, it is important to determine the concentration of this acid that will ensure good efficiency of the selective removal of iron(III) from the membrane phase. It is worth mentioning that the entire membrane process can be divided into several stages [26]:

(I) Fe(III) reacts with TBAB at the source solution/interface of PIM to form metal–carrier complexes.

(II) Metal–carrier complexes diffuse through the membrane towards the receiving phase (“the stripping solution”).

(III) At the interface of the membrane(PIM)/receiving phase, the metal–carrier complexes are dissociated by hydrogen ions and Fe(III) is released into the receiving phase.

It can be summarized that the extraction and re-extraction processes in the membrane occur simultaneously.

**Figure 6:**
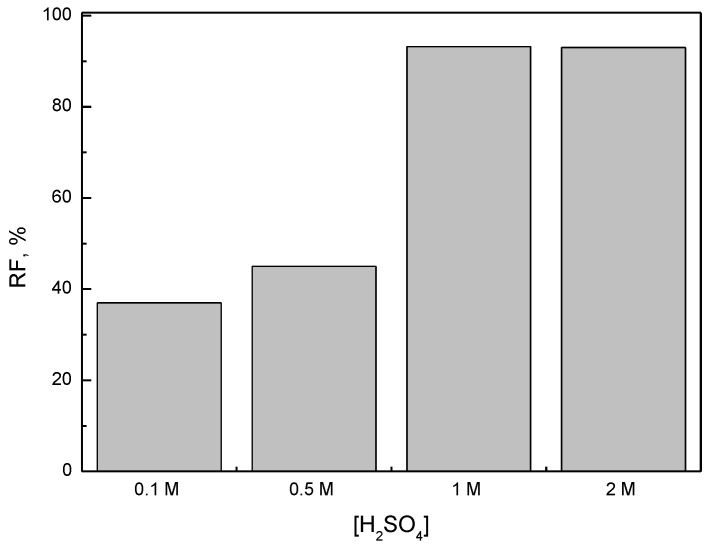
Effect of H_2_SO_4_ concentration on recovery factor (RF, %) of Fe(III). Source phase: 0.01 M Fe(III), 0.01 M Ni(II), 0.01 M Co(II), 0.01 M Li(I) in 6 M HCl solution. Receiving phase: solutions of H_2_SO_4_, PIM: 35.5% w/w CTA, 49.2% w/w NPOE, and 15.3% w/w TBAB.

Therefore, in the next study, the effect of the concentration of H_2_SO_4_ on the transport of Fe(III), Ni(II), Co(II), and Li(I) was studied. The concentration of H_2_SO_4_ was varied from 0.1 to 2 mol∙dm^−3^. Figure 6 shows the dependence of the recovery factor (*RF*) of Fe(III) at various sulfuric acid concentrations. As can be observed from Figure 6, Ni(II), Co(II), and Li(I) were not transported to the receiving phase. On the other hand, the recovery factor (*RF*) of Fe(III) rosein an increasing acid concentration up to 1 mol∙dm^−3^, and this value remained practically unchanged. Thus, based on the obtained results, it can be concluded that 1 M H_2_SO_4_ is suitable as the receiving phase in the transport of Fe(III) from 6 M HCl.

### 3.4. Structural Characterization of Polymer Inclusion Membranes with TBAB

**Figure 7:**
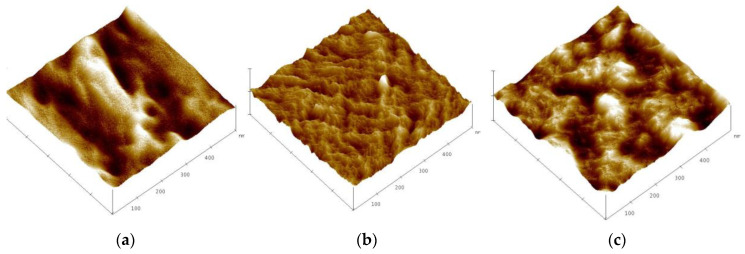
(AFM) micrographs (3D) of polymer inclusion membranes (PIMs) with TBAB: (**a**) PIM1; (**b**) PIM2; (**c**) PIM3 (composition of PIMs as in Table 2).

The surface morphology of PIM1, PIM2, and PIM3 (composition as in Table 2) was characterized by atomic force microscopy (AFM). The membranes differed in their composition. The AFM micrographs of the PIMs doped with TBAB are presented in Figure 7. As can be seen from Figure 7a–c, PIM1 is porous. This membrane has the lowest concentration of TBAB and the highest concentration of NPOE. PIM2 has the highest concentration of CTA. This membrane definitely has amore porous surface. PIM3 has the highest concentration of TBAB and the surface of this membrane is undulated. The surface morphology of PIM1, PIM2, and PIM3 appeared rough. The roughness (RMS) of PIMs in Figure 7a–c were 24.6 nm, 37.3 nm, and 35.8 nm, respectively. As can be seen, the roughness of PIMs decreases with an increasing NPOE concentration.

## 4. Discussion

The presented research results show that both the composition of the initial feed phase and the receiving phase and the composition of the membrane have a decisive influence on the efficiency of the Fe(III) ion transport process. It is worth pointing out that the appropriate composition of PIM ensures its flexibility and stability. We can conclude that a too-high concentration of TBAB used as the ion carrier adversely affects the transport of Fe(III), reducing the initial flux of the ion transfer from the source phase into the receiving phase. The conducted studies allowed the optimal TBAB concentration in PIM to be determined as 1.0 mol/dm^3^. The resulting membrane consisted of 35.5% w/w CTA, 49.2% w/w NPOE, and 15.3% w/w TBAB. We know from the literature review that ILs may have plasticizing properties. However, as can be seen from the test results, the ion carrier requires the use of an appropriate concentration of plasticizer. A too-small plasticizer content also has an adverse effect on the kinetic parameters of the process. Previous research showed that at a high concentration of ion carrier and plasticizer, we can observe the formation of a film on the membrane surface. This phenomenon causes the viscosity to increase, which limits the transport of metal ions (e.g., Fe(III)) across PIMs. These relationships are confirmed by studies by other authors [26,27,28]. On the other hand, the studies prove that the transport of metal ions through PIMs cannot occur without an ion carrier/extractant (extracting agent) in the membrane [29].

The concentration of HCl had a significant influence on the kinetic parameters of iron(III) transport and the recovery rate (*RF*) of iron(III) from the multicomponent solution. It appears that only a high concentration of HCl enables effective Fe(III) transport. It is worth noting that at high concentrations of chloride ions and HCl acid, Fe(III)occurs in the form of anion complexes of the FeCl_4_type [30,31,32] and this phenomenon probably enables the selective removal of Fe(III) by PIM with TBAB from concentrated HCl. At low concentrations of HCl, Fe(III) exists as FeCl_3_ and as cationic chlorocomplexes such as FeCl_2_^+^, FeCl^2+^, and Fe^3+^. At high concentrations of HCl, only anionic chlorocomplexes of Fe(III) (FeCl_4_^−^) easily react with TBAB at the supply phase/membrane interface. The results suggest that Fe(III) extraction with TBAB proceeds according to the anion-exchange mechanism, similar to extraction with Cyphos IL 104 [13]. Thus, it can be concluded that iron(III) is extracted to the organic phase according to the following equation:

[(Bu)_4_N]^+^_(org)_ + FeCl_4_^−^_(aq)_ ⟷ [((Bu)_4_N)FeCl_4_]_(org)_ (5)

The anionic metal chlorocomplexes of Fe(III) are extracted from the aqueous solution to the membrane phase by an anion-exchange mechanism. This mechanism is similar to the extraction of Fe(III) with other ionic liquids such as quaternary ammonium or phosphonium salts [13]. This method is also very selective towards Ni(II), Co(II), and Li(I), which do not form stable anionic chlorocomplexes in aqueous solutions in the studied conditions. Moreover, Ni(II) and Co(II) retain hexacoordination in high chloride ion concentrations in aqueous solutions [30].

On the other hand, the concentration of sulfuric acid used as the receiving phase is also a very important parameter influencing the efficiency of iron(III) transport. In previous studies [11,12,23], this acid was used as the efficient stripping phase. The stripping of Fe(III) does not linearly increase with the H_2_SO_4_ concentration because, as can be seen, this dependence is not observed for all metals. The high percentage of Fe(III) stripping from the organic phase loaded with TBAB is observed for a sufficiently high concentration of sulfuric acid determined experimentally. The concentration of acid established experimentally provides effective re-extraction of metal ions from the organic phase loaded with TBAB. In the studied condition, 1 M H_2_SO_4_ seems to be the best stripping phase for Fe(III). Many other authors have also used sulfuric acid as an effective receiving phase [1,30]. For example, Baczynska et al. [1] and Kogelnig et al. [30] confirmed that a solution of H_2_SO_4_was very effective as the stripping phase for Zn(II) transport through PIMs containing ionic liquids (ILs) as the ion carriers, such as Cyphos IL 101 (trihexyl (tetradecyl)phosphonium chloride) and Cyphos IL 104 (trihexyl(tetradecyl)phosphonium bis(2,4,4-trimethylpentyl)phosphinate).

The surface morphology of PIMs can be analyzed by AFM. As can be observed from a view of the surfaces (Figure 7), the morphologies of these membranes show pores which are filled with NPOE used as the plasticizer and TBAB used as the ion carrier. Thus, it can be concluded that cellulose triacetate membranes exhibit a porous structure, but the pores of PIM have been filled with the plasticizer molecules. Turgut et al. [33] reported that polymer membranes containing a plasticizer (e.g., NPOE), but without an ion carrier (i.e., ionic liquid), do not have a porous surface, which in turn reduces the active surface of the membrane [33]. Radzyminska-Lenarcik et al. [34] stated that the darker areas in the AFM micrographs of PIMs show pores and this may indicate crystallization of the carrier molecules inside the CTA. Arous et al. [35] reported a similar observation that PIMs are characterized by well-defined pores and these pores can be completely filled with plasticizers and carriers. Wang et al. [26] observed that higher plasticizer concentration in PIMs (e.g., NPOE) can decrease the roughness of membranes, thus improving the formation of more homogenous PIMs.

## 5. Conclusions

To date, there have been no reports in the literature on the use of TBAB as a carrier of metal ions to produce polymer membranes. In this work, TBAB was applied as the ion carrier for the synthesis of PIMs. This membrane was used for the selective separation of Fe(III) from Co(II), Ni(II), and Li(I) from an aqueous solution containing Co(II), Ni(II), and Li(I). The composition of this source phase was very similar to leach liquor of spent LIBs. The results indicate that CTA as the base polymer provides mechanical strength to PIM, NPOE improves the flexibility, and TBAB provides the polymer membrane transport and separation properties. The optimal membrane is composed of 35.5% w/w CTA, 49.2% w/w NPOE, and 15.3% w/w TBAB. A high concentration of HCl enables the effective and selective transport of Fe(III) from the source phase into 1 M H_2_SO_4_. This method can be employed to remove iron(III) impurities from solutions with Ni(II), Co(II), and Li(I).

## Figures and Tables

**Table 1:** Kinetic parameters of Fe(III) transport depending on HCl concentration in source phase. Conditions are as in Figure 2.

HCl Concentration, mol·dm^−3^	Rate Constant, h^−1^	Permeability Coefficient, P, µmol·s^−1^
1.0	0.0157	0.348
3.0	0.0290	0.640
6.0	0.1650	3.650

**Table 2:** Composition of PIMs.

No	Polymer Support, CTA, (wt.%)	Plasticizer NPOE (wt.%)	Ion Carrier TBAB (wt.%)	Ion Carrier Concentration [M]
PIM1:	35.5	49.2	15.3	1.0
PIM2:	47.1	32.7	20.2	1.5
PIM3:	33.3	34.7	32.0	2.0

## Data Availability

Not applicable.
